# Supplementary material for: Impairment of a distinct cancer-associated fibroblast population limits tumour growth and metastasis
Source: Nat Commun. 2021 Jun 10;12:3516. doi: 10.1038/s41467-021-23583-1 (PMC8192501; doi:10.1038/s41467-021-23583-1)
Supplement: Supplementary file 1 — Supplementary Information [file 41467_2021_23583_MOESM1_ESM.pdf]

Impairment of a distinct cancer-associated fibroblast population limits tumour growth and metastasis

Ute Jungwirth, Antoinette van Weverwijk, Rachel J. Evans, Liam Jenkins, David Vicente, John Alexander, Qiong Gao, Syed Haider, Marjan Iravani and Clare M. Isacke

SUPPLEMENTARY MATERIAL

Supplementary methods

Supplementary Tables 1 - 6

Supplementary Table 1a. | Top 30 upregulated pathways in D2A1-m12 cells compared to D2A1 cells
Supplementary Table 1b. | Top 30 downregulated pathways in D2A1-m12 cells compared to D2A1 cells
Supplementary Table 2 | Top 50 upregulated matrisome genes in D2A1-m12 cells versus D2A1 cells
Supplementary Table 3 | Antibodies
Supplementary Table 4 | Mission shRNA lentiviral particles targeting Endo180 (*Mrc2*) (Sigma)
Supplementary Table 5 | ON-TARGET *plus* siRNA targeting Endo180 (*Mrc2*) (Dharmacon)
Supplementary Table 6 | Taqman PCR primers

Supplementary Figures 1 - 9

Supplementary Fig. 1 | Fibroblast expression of Endo180 in solid tumours
Supplementary Fig. 2 | Endo180 promotes metastatic tumour growth in lungs and liver
Supplementary Fig. 3 | Additional *in vivo* models
Supplementary Fig. 4 | Endo180 expression following siRNA- or shRNA-mediated knockdown
Supplementary Fig. 5 | Fibroblast-tumour cell co-culture assays
Supplementary Fig. 6 | Cell spreading on collagen or fibronectin-coated hydrogels
Supplementary Fig. 7 | Fibroblast contractility markers
Supplementary Fig. 8 | Comparison of the parental D2A1 line and the D2A1-m12 subline
Supplementary Fig. 9 | Gene expression analysis of the D2A1 line and the D2A1-m12 subline

Supplementary material references

Supplementary methods

Whole exome sequencing

DNA was extracted from tumour cell lines or BALB/c mouse spleen tissue using the DNeasy Blood and Tissue kit. DNA samples were physically sheared to the desired size using a Covaris E Series instrument (Covaris). Paired-end multiplexed library preparation was performed by the Tumour Profiling Unit at the ICR using the SureSelect^{XT} Library Prep and Capture System (Agilent Technologies) following the standard protocol workflow, before multiplex sequencing on a NovaSeq 6000 flow cell (Illumina).

Bioinformatics and statistical analyses were performed using custom R scripts and a bespoke DNA sequencing pipeline based on the Nextflow framework[1]. Whole exome sequencing FASTQ files were aligned to the mouse genome assembly build GRCm38 with the Burrows-Wheeler Aligner (BWA) v0.7.12[2]. BWA was run with default parameters utilising 5 threads. The resulting SAM file was converted to BAM and sorted with SAMtools v1.5[3]. Duplicate reads were removed from the sample files with the Picard v2.8.1 suite of tools (http://broadinstitute.github.io/picard/). Insert size and coverage metrics were also calculated with Picard. Lastly, base scores were recalibrated with the Genome Analysis Toolkit (GATK) v4.0.3.0[4] according to the GATK Best Practices pipeline.

Copy number analysis (CNA) was carried out with CNVkit v0.9.3[5], utilising the batch command. A cancer-free sample taken from BALB/c mouse spleen tissue was used as a normal reference for each cell line sample. Copy number $\log_2$ ratios from CNVkit were used to create CNA plots in R statistical programming language v3.5.0 (R Core Team, 2018).

LoFreq[6] was used to call somatic variants. In CNA, the same healthy BALB/c sample was used as a normal reference. Common variants were removed before annotating the remaining variants with ANNOVAR (01/02/2016 release)[7]. The output from ANNOVAR was further processed in R for comparisons between samples and visualisations.

Supplementary Tables

Supplementary Table 1a. | Top 30 upregulated pathways in D2A1-m12 cells compared to D2A1 cells

Pathway	NES	FDR
mouse.corematrisome.collagens	1.886	0.038
mouse.matrisome.associated.ecm.affiliated	1.869	0.019
mouse.corematrisome.glycoproteins	1.836	0.024
BIOCARTA_MM_CASPASE_CASCADE_IN_APOPTOSIS	1.793	0.058
BIOCARTA_MM_THROMBIN_SIGNALING_AND_PROTEASE-ACTIVATED_RECEPTORS	1.792	0.038
BIOCARTA_MM_D4-GDI_SIGNALING_PATHWAY	1.732	0.038
PANTHER_MM_INTEGRIN_SIGNALLING_PATHWAY	1.729	0.019
BIOCARTA_MM_RHO_CELL_MOTILITY_SIGNALING_PATHWAY	1.633	0.224
mouse.matrisome.associated.regulators	1.582	0.079
PANTHER_MM_HETEROTRIMERIC_G-PROTEIN_SIGNALING_PATHWAY-GI_ALPHA_AND_GS_ALPHA_MEDIATED	1.562	0.179
PANTHER_MM_FAS_SIGNALING_PATHWAY	1.548	0.345
BIOCARTA_MM_PHOSPHOLIPIDS_AS_SIGNALLING_INTERMEDIARIES	1.540	0.353
BIOCARTA_MM_RAC_1_CELL_MOTILITY_SIGNALING_PATHWAY	1.533	0.371
BIOCARTA_MM_INTEGRIN_SIGNALING_PATHWAY	1.525	0.345
BIOCARTA_MM_RAS-INDEPENDENT_PATHWAY_IN_NK_CELL-MEDIATED_CYTOTOXICITY	1.522	0.371
PANTHER_MM_IONOTROPIC_GLUTAMATE_RECEPTOR_PATHWAY	1.511	0.371
BIOCARTA_MM_ROLES_OF_ARRESTIN-DEPENDENT_RECRUITMENT_OF_SRC_KINASES_IN_GPCR_SIGNALING	1.493	0.393
PANTHER_MM_INFLAMMATION_MEDIATED_BY_CHEMOKINE_AND_CYTOKINE_SIGNALING	1.489	0.156
BIOCARTA_MM_ROLE_OF_ARRESTINS_IN_THE_ACTIVATION_AND_TARGETING_OF_MAP_KINASES	1.477	0.399
BIOCARTA_MM_SPLICEOSOMAL_ASSEMBLY	1.470	0.399
BIOCARTA_MM_CCR3_SIGNALING_IN_EOSINOPHILS	1.467	0.403
BIOCARTA_MM_TGF_BETA_SIGNALING_PATHWAY	1.464	0.399
NETPATH_MM_ALPHA6_BETA4_INTEGRIN	1.423	0.371
NETPATH_MM_EGFR1_SIGNALING_PATHWAY	1.415	0.038
mouse.matrisome.associated.secretedfactors	1.409	0.345
PANTHER_MM_CYTOSKELETAL_REGULATION_BY_RHO_GTPASE	1.408	0.393
NETPATH_MM_B_CELL_RECEPTOR_SIGNALING_PATHWAY	1.403	0.314
PANTHER_MM_HETEROTRIMERIC_G-PROTEIN_SIGNALING_PATHWAY-GQ_ALPHA_AND_GO_ALPHA_MEDIATED	1.382	0.421
PANTHER_MM_HUNTINGTON_DISEASE	1.377	0.371
BIOCARTA_MM_MTOR_SIGNALING_PATHWAY	1.357	0.602
BIOCARTA_MM_TREFOIL_FACTORS_INITIATE_MUCOSAL_HEALING	1.352	0.602

Pathways highlighted in yellow have FDR < 0.1. FDR, false discovery rate. NES, normalised enrichment score

Supplementary Table 1b. | Top 30 downregulated pathways in D2A1-m12 cells compared to D2A1 cells		
Pathway	NES	FDR
BIOCARTA_MM_SYNAPTIC_PROTEINS_AT_THE_SYNAPTIC_JUNCTION	-1.606	0.345
PANTHER_MM_SYNAPTIC_VESICLE_TRAFFICKING	-1.531	0.391
PANTHER_MM_INSULIN_IGF_PATHWAY-PROTEIN_KINASE_B_SIGNALING_CASCADE	-1.527	0.371
PANTHER_MM_METABOTROPIC_GLUTAMATE_RECEPTOR_GROUP_I_PATHWAY	-1.429	0.558
BIOCARTA_MM_BONE_REMODELLING	-1.404	0.602
BIOCARTA_MM_REGULATION_OF_EIF2	-1.400	0.600
PANTHER_MM_EGF_RECEPTOR_SIGNALING_PATHWAY	-1.321	0.393
BIOCARTA_MM_TELOMERES_TELOMERASE_CELLULAR_AGING_AND_IMMORTALITY	-1.299	0.646
PANTHER_MM_MUSCARINIC_ACETYLCHOLINE_RECEPTOR_1_AND_3_SIGNALING_PATHWAY	-1.272	0.614
BIOCARTA_MM_TOLL-LIKE_RECEPTOR_PATHWAY	-1.271	0.602
PANTHER_MM_P53_PATHWAY_FEEDBACK_LOOPS_2	-1.269	0.602
PANTHER_MM_CORTOCOTROPIN_RELEASING_FACTOR_RECEPTOR_SIGNALING_PATHWAY	-1.264	0.667
PANTHER_MM_PI3_KINASE_PATHWAY	-1.259	0.602
BIOCARTA_MM_CD40L_SIGNALING_PATHWAY	-1.248	0.704
PANTHER_MM_PDGF_SIGNALING_PATHWAY	-1.222	0.568
PANTHER_MM_HISTAMINE_H1_RECEPTOR_MEDIATED_SIGNALING_PATHWAY	-1.205	0.692
PANTHER_MM_INTERLEUKIN_SIGNALING_PATHWAY	-1.188	0.656
BIOCARTA_MM_INHIBITION_OF_CELLULAR_PROLIFERATION_BY_GLEEVEC	-1.186	0.757
PANTHER_MM_INSULIN_IGF_PATHWAY-MITOGEN_ACTIVATED_PROTEIN_KINASE_KINASE_MAP_KINASE_CASCADE	-1.177	0.753
BIOCARTA_MM_P53_SIGNALING_PATHWAY	-1.094	0.808
PANTHER_MM_OXIDATIVE_STRESS_RESPONSE	-1.092	0.795
BIOCARTA_MM_P38_MAPK_SIGNALING_PATHWAY_	-1.087	0.795
BIOCARTA_MM_KERATINOCYTE_DIFFERENTIATION	-1.087	0.795
BIOCARTA_MM_SKELETAL_MUSCLE_HYPERTROPHY_IS_REGULATED_VIA_AKT_MTOR_PATHWAY	-1.083	0.808
PANTHER_MM_HYPOXIA_RESPONSE_VIA_HIF_ACTIVATION	-1.067	0.808
BIOCARTA_MM_THE_4-1BB-DEPENDENT_IMMUNE_RESPONSE	-1.052	0.827
PANTHER_MM_OXYTOCIN_RECEPTOR_MEDIATED_SIGNALING_PATHWAY	-1.050	0.824
PANTHER_MM_5HT2_TYPE_RECEPTOR_MEDIATED_SIGNALING_PATHWAY	-1.031	0.827
PANTHER_MM_HISTAMINE_H2_RECEPTOR_MEDIATED_SIGNALING_PATHWAY	-1.023	0.872
BIOCARTA_MM_NEUROPEPTIDES_VIP_AND_PACAP_INHIBIT_THE_APOPTOSIS_OF_ACTIVATED_T_CELLS	-0.987	0.922
BIOCARTA_MM_CERAMIDE_SIGNALING_PATHWAY	-0.970	0.922
FDR, false discovery rate. NES, normalised enrichment score		

Supplementary Table 2 | Top 50 upregulated matrisome genes in D2A1-m12 cells versus D2A1 cells

Gene	Matrisome.Geneset	logFC	FDR
Ngf	matrisome.associated.secretedfactors	10.84	1.17E-06
Tnfsf13b	matrisome.associated.secretedfactors	9.45	1.72E-06
Lamb3	corematrisome.glycoproteins	7.44	1.34E-03
Col6a1	corematrisome.collagens	6.73	1.40E-09
Thbs2	corematrisome.glycoproteins	6.46	1.82E-04
Fcna	matrisome.associated.ecm.affiliated	5.83	2.07E-03
Fam20a	matrisome.associated.regulators	5.64	3.12E-05
Igfbp6	corematrisome.glycoproteins	5.57	3.46E-10
Podnl1	corematrisome.proteoglycans	5.51	3.46E-10
Ccl2	matrisome.associated.secretedfactors	5.50	3.71E-06
Col1a1	corematrisome.collagens	5.39	2.92E-07
Col6a2	corematrisome.collagens	5.24	2.37E-05
Nid2	corematrisome.glycoproteins	5.15	1.03E-03
Adam3	matrisome.associated.regulators	4.85	1.64E-02
Ccl7	matrisome.associated.secretedfactors	4.68	9.57E-03
Col6a3	corematrisome.collagens	4.54	1.18E-04
Slit1	corematrisome.glycoproteins	4.38	9.33E-08
Serpinf1	matrisome.associated.regulators	4.38	3.06E-05
Ctsh	matrisome.associated.regulators	4.34	5.14E-06
C1qtnf6	matrisome.associated.ecm.affiliated	4.26	3.62E-09
Wnt10b	matrisome.associated.secretedfactors	4.25	2.87E-05
Col3a1	corematrisome.collagens	4.25	6.13E-06
Sema4a	matrisome.associated.ecm.affiliated	4.24	9.70E-06
Ntng2	corematrisome.glycoproteins	4.17	3.24E-05
S100a16	matrisome.associated.secretedfactors	4.14	1.91E-06
Il34	matrisome.associated.secretedfactors	4.13	4.72E-07
Col5a3	corematrisome.collagens	4.10	3.52E-03
Col18a1	corematrisome.collagens	3.86	6.75E-08
Angptl4	matrisome.associated.secretedfactors	3.67	1.35E-04
Lama3	corematrisome.glycoproteins	3.63	9.22E-06
Wnt1	matrisome.associated.secretedfactors	3.61	3.35E-04
Fgf1	matrisome.associated.secretedfactors	3.57	1.24E-04
Mst1	matrisome.associated.secretedfactors	3.44	6.04E-04
Anxa8	matrisome.associated.ecm.affiliated	3.23	1.81E-04
Lgi3	corematrisome.glycoproteins	3.13	8.45E-03
Mmp28	matrisome.associated.regulators	3.03	2.11E-03
Sema7a	matrisome.associated.ecm.affiliated	3.02	1.96E-04
Emilin1	corematrisome.glycoproteins	3.00	6.75E-08
Loxl3	matrisome.associated.regulators	2.78	7.86E-07
Elfn2	matrisome.associated.ecm.affiliated	2.74	1.49E-01
Sema6b	matrisome.associated.ecm.affiliated	2.73	3.20E-07
Loxl2	matrisome.associated.regulators	2.73	2.49E-02
Htra3	matrisome.associated.regulators	2.69	1.33E-03
Fgfbp3	matrisome.associated.secretedfactors	2.62	9.83E-07
C1qtnf1	matrisome.associated.ecm.affiliated	2.60	1.00E-04
Ctso	matrisome.associated.regulators	2.54	1.34E-04
Adam8	matrisome.associated.regulators	2.44	9.97E-05
Cspg4	matrisome.associated.ecm.affiliated	2.43	5.84E-03
Dhh	matrisome.associated.secretedfactors	2.37	3.38E-03
Pdgfa	matrisome.associated.secretedfactors	2.31	6.75E-07

FDR, false discovery rate; LogFC, log fold change

Supplementary Table 3 | Antibodies				
**Antibody**	**Species**	**Source (catalogue number)**	**Assays**	**Dilution**
αSMA (clone1A4)	mouse	Sigma (A2547)	IHC<br>IF	1:1,000<br>1:4,000
anti-mouse CD16/CD31	rat	eBioscience (14-0161)	Fc receptor block for FACS	1:100
CD24	rat	eBioscience (M1/69)	Magnetic sorting	1:250
CD31 (clone 39, FITC conjugated)	rat	BioLegend (102406)	FACS	1:100
CD45 (30-F11, PE/Cy5 conjugated)	rat	BD Biosciences (553082)	FACS	1:500
CD45	rat	R&D Systems (MAB114)	Magnetic sorting	1:250
Endo180 (human)	mouse	mAb 39.10 (in house) [8]	IHC	1:1,000
Endo180 (mouse)	sheep	R&D Systems (AF4789)	WB<br>IHC	1:1,000<br>1:100
Endomucin (clone V.7C7)	rat	Santa Cruz (SC65495)	IHC	1:1,000
F4/80 (clone CI:A3-1, AF647 conjugated)	rat	Bio-Rad (MCA497A647)	FACS	1:100
F4/80 (clone CI:A3-1)	rat	Bio-Rad (MCA497G)	IHC	1:1,00
Fibronectin	rabbit	Dako (A0245)	IF	1:2,000
GFP	Rabbit	Invitrogen (A11122)	IHC	1:2,000
MLC	rabbit	Cell Signaling (3672)	WB	1:1,000
p(Ser19)-MLC	mouse	Cell Signaling (3675)	WB<br>IF	1:500<br>1:50
MYPT	rabbit	Cell Signaling (2634)	WB	1:500
p(Thr696)-MYPT	rabbit	Cell Signaling (5163)	WB	1:500
PDGFRα (clone APA5, BV605 conjugated)	rat	BioLegend (135916)	FACS	1:200
Tubulin	mouse	Sigma (T5168)	WB	1:10,000
YAP (clone 63.7)	mouse	Santa Cruz (sc-101199)	IF	1:50
Alexa488-phalloidin	N/A	Molecular Probes (A12379)	IF	1:500
Alexa555-phalloidin	N/A	Molecular Probes (A34055)	IF	1:500
DAPI	N/A	Molecular Probes (D1306)	IF	1:10,000
IgG-HRP-anti-mouse	donkey	Santa Cruz (sc-2314)	WB	1:10,000
IgG-HRP-anti-rabbit	goat	Santa Cruz (sc-2004)	WB	1:10,000
IgG-HRP-anti-sheep	rabbit	Santa Cruz (sc-2770)	WB	1:10,000

FACS, fluorescence activated cell sorting; IF, immunofluorescence; IHC, immunohistochemistry; WB, western blot.

**Supplementary Table 4 | Mission shRNA lentiviral particles targeting Endo180 (*Mrc2*) (Sigma)**		
**Clone ID**	**Gene target**	**NM ID**
SHC002V	NTC	N/A
TRCN00001239-25	*Mrc2*	NM_008626.3
TRCN00001239-27	*Mrc2*	NM_008626.3
TRCN00001239-28	*Mrc2*	NM_008626.3

**Supplementary Table 5 | ON-TARGET *plus* siRNA targeting Endo180 (*Mrc2*) (Dharmacon)**		
**ID**	**Gene target**	**NM ID**
D-001810-01	NTC	N/A
D-001810-04	NTC	N/A
J-040940-09	*Mrc2*	NM_008626.3
J-040940-12	*Mrc2*	NM_008626.3

Supplementary Table 6 | Taqman® gene expression probes, Life Technologies

Probe ID	Gene symbol	Species	Reporter
Mm00725412_s1	*Acta2*	Mouse	FAM
Mm00802529_m1	*Adgre1*	Mouse	FAM
Mm00437762_m1	*B2m*	Mouse	FAM
Mm01191753_m1	*Bgn*	Mouse	FAM
Mm00801666_g1	*Col1a1*	Mouse	FAM
Mm00483888_m1	*Col1a2*	Mouse	FAM
Mm00489842_m1	*Col5a3*	Mouse	FAM
Mm00493214_m1	*Epcam*	Mouse	FAM
Mm01329177_m1	*Fap*	Mouse	FAM
Mm00599696_m1	*Igfbp6*	Mouse	FAM
Mm04209403_g1	*Krt8*	Mouse	FAM
Mm01254735_m1	*Lama3*	Mouse	FAM
Mm01307379_m1	*Ltbp2*	Mouse	FAM
Mm01251581_m1	*Mrc2*	Mouse	FAM
Mm01251442_m1	*Myl9*	Mouse	FAM
Mm01325566_m1	*Ntng2*	Mouse	FAM
Mm00440701_m1	*Pdgfra*	Mouse	FAM
Mm01242576_m1	*Pecam1*	Mouse	FAM
Mm01293577_m1	*Ptprc*	Mouse	FAM
Mm01279240_m1	*Thbs2*	Mouse	FAM
Mm01201237_m1	*Ubc*	Mouse	FAM

Supplementary Fig. 1

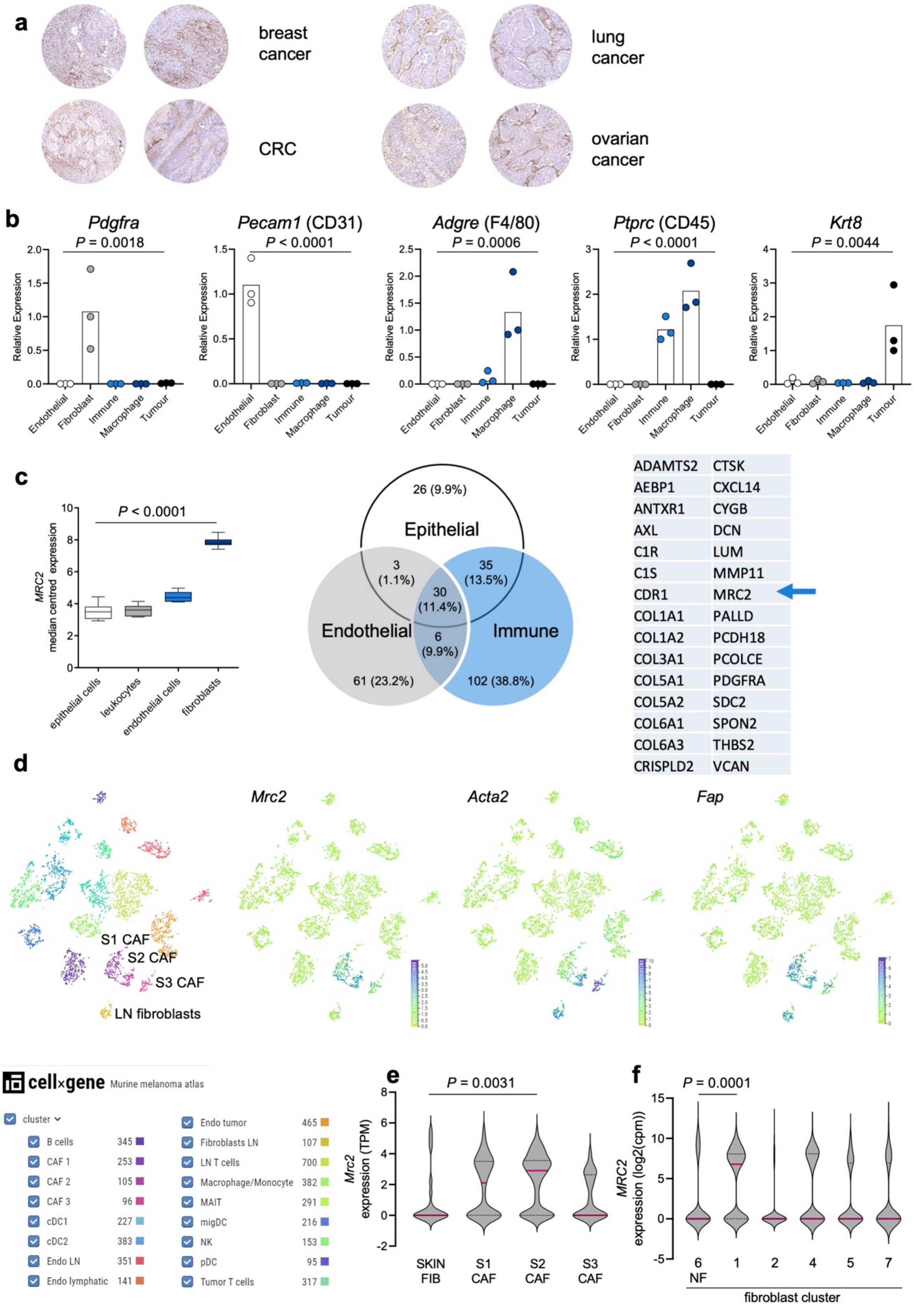

ADAMTS2	CTSK
AEBP1	CXCL14
ANTXR1	CYGB
AXL	DCN
C1R	LUM
C1S	MMP11
CDR1	MRC2
COL1A1	PALLD
COL1A2	PCDH18
COL3A1	PCOLCE
COL5A1	PDGFRA
COL5A2	SDC2
COL6A1	SPON2
COL6A3	THBS2
CRISPLD2	VCAN

Supplementary Fig. 1 | Fibroblast expression of Endo180 in solid tumours

a Images taken from the Human Protein Atlas (www.proteinatlas.org, version 19.0)[9] of breast, colorectal (CRC), lung and ovarian tumours stained for Endo180. Related to Fig.1a, where a comparable pattern of Endo180 staining in human breast cancers is observed using an independent antibody. **b** Relative expression of *Pdgfra*, *Pecam1* (CD31), *Adgre1* (F40/80), *Ptprc* (CD45) and *Krt8* in endothelial cells, fibroblasts, immune cells (CD45+/F4/80-), macrophages (F4/80+) and tumour cells isolated by FACS sorting from 3 independent D2A1-m2 tumours (mean values, one-way ANOVA). Related to Fig. 1b. **c** Endo180 (*MRC2*) expression in epithelial cells, leucocytes, endothelial cells and fibroblasts purified from human colorectal cancers (n=6 tumours) (GSE39397[10]). Left panel, box plots shows median and 25th to 75th quartiles, whiskers show minimum and maximum (one-way ANOVA with Tukey's multiple comparison test). Middle panel, upregulated expression of genes with P value < 10^{-11} in CAFs compared to endothelial, immune and/or epithelial/tumour cell populations. Right panel, list of the 30 genes commonly upregulated in CAF *vs.* endothelial, CAF *vs.* immune and CAF *vs.* epithelial/tumour cells, with Endo180 (*MRC2*) indicated. **d** tSNE visualisation of colour coded cell types isolated from primary B16-F10 mouse melanoma tumours and draining lymph nodes and subject to single cell RNA-seq by Davidson and colleagues[11]. Shown are expression plots for *Mrc2*, *Acta2* and *Fap* of tumour and lymph node tissue of tumour bearing mice. Data was visualised using the online tool provided by the authors (https://melanoma.cellgeni.sanger.ac.uk/). **e** Using the same dataset, quantification of *Mrc2* fibroblast expression in control normal skin (n=20) and the fibroblast subpopulations from tumours; CAF S1 (n=124), CAF S2 (n=229) and CAF S3 (n=93). Red bars indicate median expression levels, dotted lines indicate upper and lower quartiles (Kruskal-Wallis test, Dunn's correction). **f** Equivalent analysis of the Lambrechts and colleagues single cell RNA-seq data from human lung tumours and non-malignant lung tissue[12]. Gene expression values were exported from the Scope online tool (https://gbiomed.kuleuven.be/scRNAseq-NSCLC). Violin plots show *MRC2* expression in the 6 fibroblast clusters defined by the authors. Clusters 6 represents normal fibroblasts (n=175), Clusters 1 (n=315), 2 (n=321), 4 (n=219), 5 (n=195), 7 (n=55) represent tumour associated-fibroblasts (Kruskal-Wallis test, Dunn's correction). Source data are provided as a Source Data file.

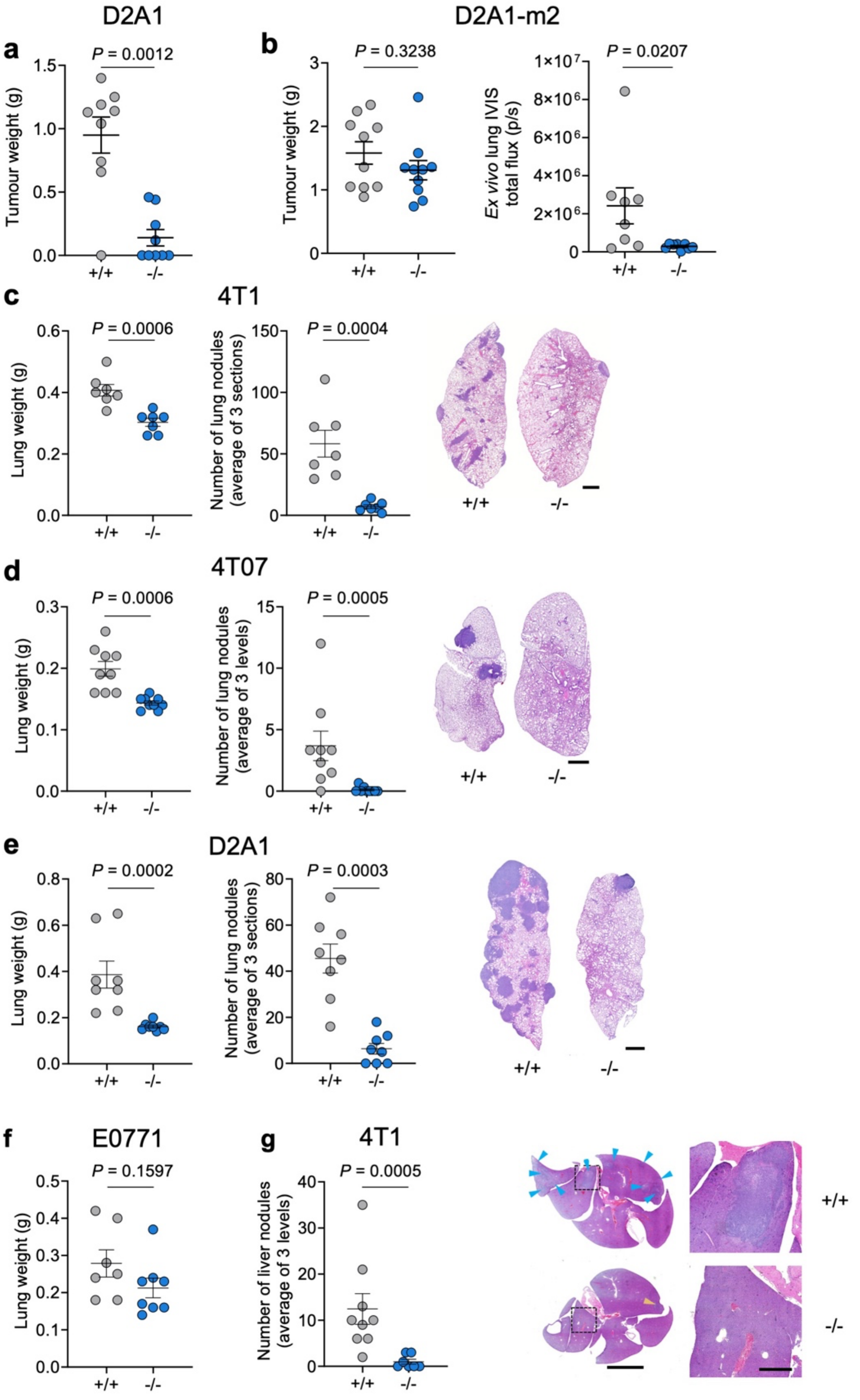

D2A1
D2A1-m2
a
b
P = 0.0012
Tumour weight (g)
P = 0.3238
Tumour weight (g)
P = 0.0207
Ex vivo lung IVIS total flux (p/s)
+/+
-/-
c
4T1
P = 0.0006
Lung weight (g)
P = 0.0004
Number of lung nodules (average of 3 sections)
d
4T07
P = 0.0006
Lung weight (g)
P = 0.0005
Number of lung nodules (average of 3 levels)
e
D2A1
P = 0.0002
Lung weight (g)
P = 0.0003
Number of lung nodules (average of 3 sections)
f
E0771
P = 0.1597
Lung weight (g)
g
4T1
P = 0.0005
Number of liver nodules (average of 3 levels)

Supplementary Fig. 2 | Endo180 promotes metastatic tumour growth in lungs and liver
Additional quantification of the data presented in Fig. 2. All data are mean values ±SEM and either two-sided *t*- or Mann-Whitney *U* tests, as indicated. Quantification of metastatic nodules represents mean number of lung nodules in 3 lung or liver sections. **a** D2A1 cells inoculated into the 4th mammary fat pad from Figure 2c. Shown are final tumour weights at day 39 (n=9 per group, Mann-Whitney *U* test). **b** D2A1-m2-Luc cells inoculated into the 4th mammary fat pad from Fig. 2d. Shown are final primary tumour weights at day 40 (left graph, n=10 per group, Mann-Whitney *U* test) and quantification of spontaneous metastasis to the lung by *ex vivo* IVIS imaging (right graph, n=8 per group, Mann-Whitney *U* test). **c** 4T1-Luc cells injected intravenously into BALB/c mice from Fig. 2e (n=7 per group). Shown are *ex vivo* lung weights (*t*-test), number of metastatic lung nodules (*t*-test) and representative H&E stained lung sections (scale bar, 1 mm). **d** 4T07 cells injected intravenously into BALB/c mice from Fig. 2f (n=9 per group). Shown are *ex vivo* lung weights (*t*-test), number of metastatic lung nodules (Mann-Whitney *U* test) and representative H&E stained sections (scale bar, 1 mm). **e** D2A1 cells injected intravenously into BALB/c mice from Fig. 2g (n=8 per group). Shown are *ex vivo* lung weights (Mann-Whitney *U* test), number of metastatic lung nodules (*t*-test) and representative H&E stained sections (scale bar, 1 mm). **f** E0771-Luc cells injected intravenously into C57BL/6 mice from Fig. 2i. Shown are *ex vivo* lung weights (Endo180$^{+/+}$, n=7; End180$^{-/-}$, n=8; *t*-test). **g** 4T1-Luc cells injected into the spleen parenchyma of BALB/c mice from Fig. 2j. Shown are number of metastatic liver nodules (Endo180$^{+/+}$ n=9, Endo180$^{-/-}$ n=7, Mann-Whitney *U* test) and low (scale bar, 5 mm) and high (scale bar, 1 mm) power images of H&E stained liver sections. Arrowheads indicate tumour nodules. Source data are provided as a Source Data file.

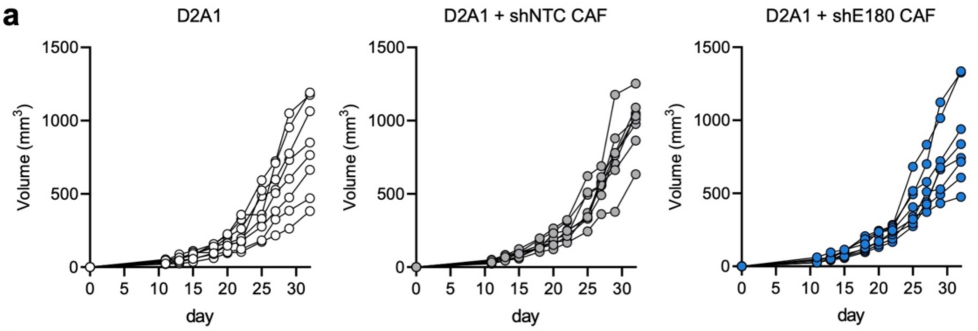

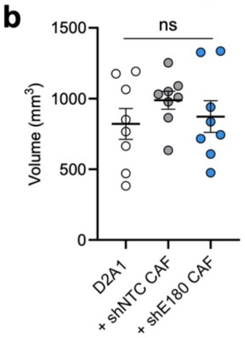

	D2A1	+ shNTC CAF	+ shE180 CAF
Δ volume (mm^3) between largest and smallest tumour	809.4	618.3	860.1

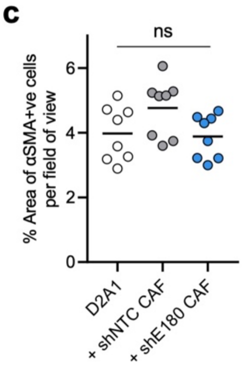

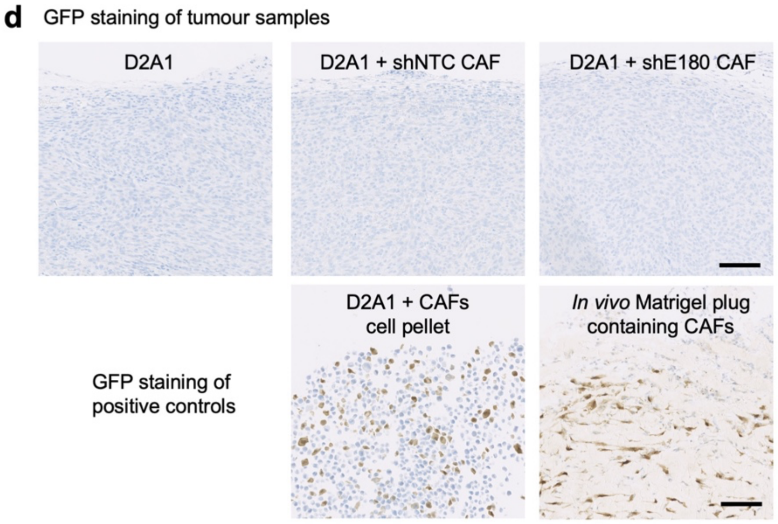

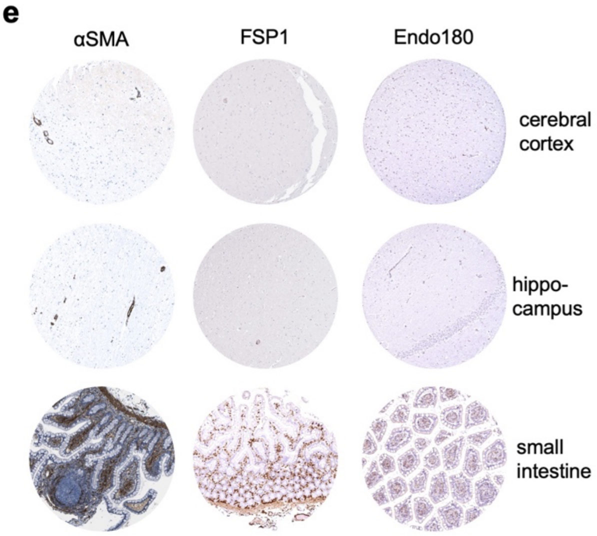

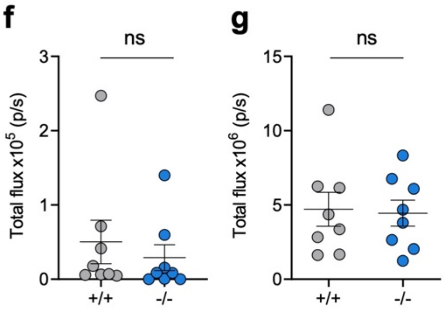

Supplementary Fig. 3 | Additional *in vivo* models

a 2 x 10^5 D2A1 tumour cells were injected alone (left panel) or admixed with 6 x 10^5 shNTC (middle panel) or shE180 (right panel) GFP-positive CAFs orthotopically into the 4th mammary fat pad of BALB/c mice. Shown are; primary tumour growth in individual mice. **b** Left graph, final tumour volume at day 32 (n=8 per group, mean values ±SEM, one-way ANOVA). Right panel, difference between largest and smallest final tumour volume. **c** Analysis of αSMA positive staining of primary tumours. Data shown represent mean values ±SEM (n=8 per group, one-way ANOVA). **d** GFP staining of IHC sections. Top row, representative tumour samples from panel a. Equivalent images were obtained in an additional independent experiment. Bottom row, positive control samples. Left panel, section from a FFPE cell pellet of GFP-negative D2A1 cells mixed with GFP-positive CAFs. Right panel, section of FFPE Matrigel plug containing GFP-positive CAFs inoculated into the flank of a BALB/c mouse and removed after 7 days (scale bars, 100 μm). Equivalent images were collected in 3 different Magrigel plugs. Further, no GFP staining was seen in plugs containing GFP-negative CAFs. **e** Images taken from The Human Protein Atlas (http://www.proteinatlas.org, version 19.0)[9] of brain (cerebral cortex and hippocampus) and small intestine stained for Endo180 and the fibroblast markers αSMA and FSP1. **f** 2.5 x 10^3 4T1-Luc cells were injected intracranially (supraventorial) into BALB/c mice. Metastatic colonisation was measured by *ex vivo* IVIS imaging on day 12 (n=8 per group, mean values ±SEM, two-sided *t*-test, no significant differences). **g** 3 x 10^5 4T1-Luc cells were injected into the left ventricle of the heart and metastasis to the brain monitored by *in vivo* IVIS imaging on day 10 (n=8 per group, mean values ±SEM, two-sided *t*-test, no significant differences). Source data are provided as a Source Data file.

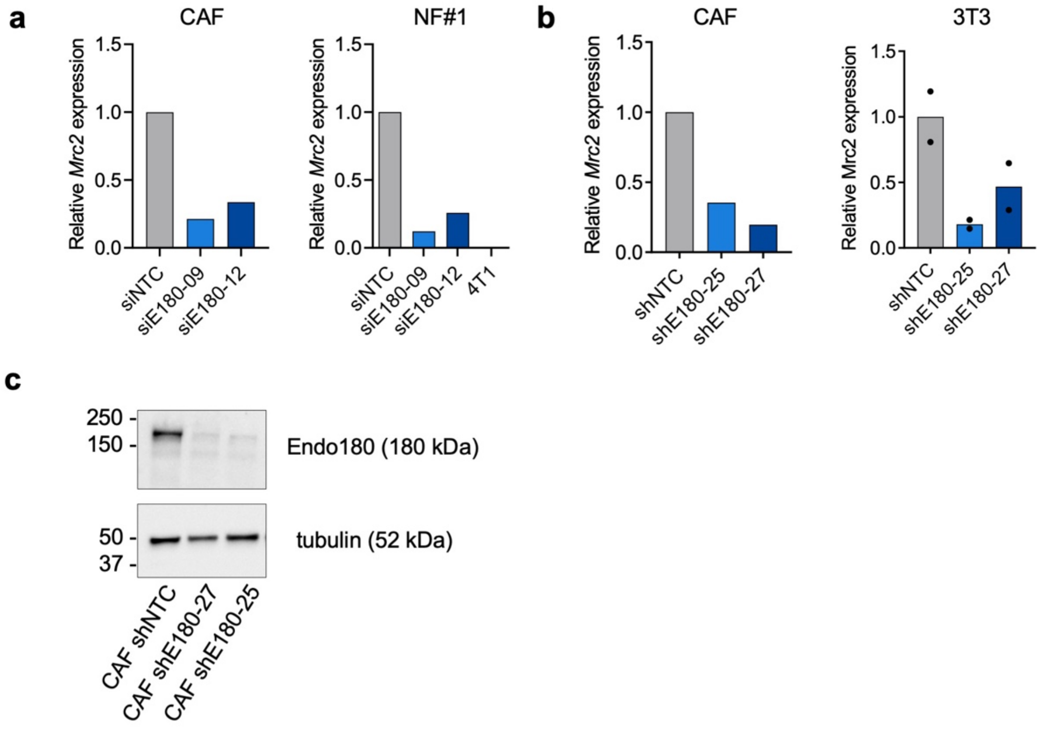

Supplementary Fig. 4 | Endo180 expression following siRNA- or shRNA-mediated knockdown

a, b Relative Endo180 (*Mrc2*) expression in CAFs and in NF#1 and 3T3 mouse fibroblasts after transfection/transduction with two independent siRNAs (panel a) or shRNAs (panel b) in comparison to non-targeting control (NTC) analysed by RT-qPCR (panel b; 3T3, n=2). No expression was detected in 4T1 tumour cells. Equivalent results were obtained in at least three independent experiments. **c** Western blot of Endo180 (180 kDa) in CAFs transduced with either non-targeting control shRNA (shNTC) or two independent shRNAs against Endo180. Tubulin serves as loading control. Molecular size markers are in kDa. Western blot analysis was replicated with the same, but also additional, cell lines in at least 3 independent experiments (see also Fig. 5b). Source data are provided as a Source Data file.

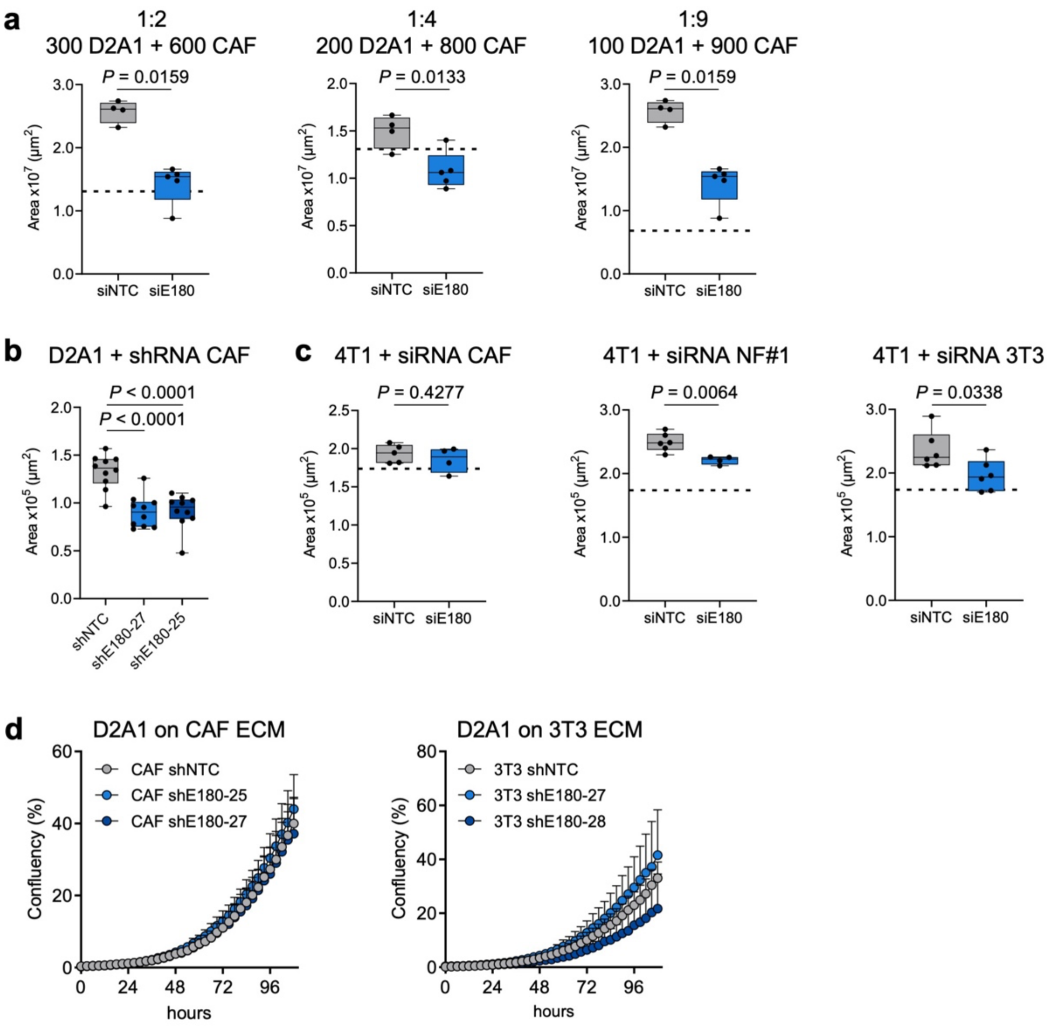
a
1:2
300 D2A1 + 600 CAF
P = 0.0159
1:4
200 D2A1 + 800 CAF
P = 0.0133
1:9
100 D2A1 + 900 CAF
P = 0.0159
Area x10^7 (μm^2)
siNTC
siE180
b
D2A1 + shRNA CAF
P < 0.0001
P < 0.0001
Area x10^5 (μm^2)
shNTC
shE180-27
shE180-25
c
4T1 + siRNA CAF
P = 0.4277
4T1 + siRNA NF#1
P = 0.0064
4T1 + siRNA 3T3
P = 0.0338
d
D2A1 on CAF ECM
CAF shNTC
CAF shE180-25
CAF shE180-27
D2A1 on 3T3 ECM
3T3 shNTC
3T3 shE180-27
3T3 shE180-28
Confluency (%)
hours

Supplementary Fig. 5 | Fibroblast-tumour cell co-culture assays

In all experiments, fibroblasts were transfected/transduced with non-targeting control (NTC) or Endo180 si/shRNAs and box plots in a-c show median and 25th to 75th quartiles, whiskers show minimum and maximum. **a** Different ratios of D2A1 tumour cells and siRNA transfected CAFs were co-cultured in U-bottom low adherence plates for 8 days. Mean size of D2A1 spheroids alone is indicated by the dotted line (n=4, siNTC group: n=5 for siE180 group, 1:3 ratio, two-sided Mann-Whitney *U* test; 1:5 ratio, two-sided *t*-test; 1:9 ratio, two-sided Mann-Whitney *U* test). Related to Fig. 3a. **b** Equivalent results to panel a were obtained using shRNA transduced CAFs. Shown are 1:3 ratio co-cultures of D2A1 and CAFs transduced with non-targeting (NTC) or two independent Endo180-targeting shRNAs after 6 days (n=10 spheroids per condition; mean values ±SEM, one-way ANOVA). Related to Fig. 3a. **c** 4T1 cells admixed with CAFs (n=5, siNTC group; n=4, siE180 group), NF#1 (n=6, siNTC group; n=4 siE180 group) or 3T3 (n=6, siNTC group; n=6 siE180 group) fibroblasts in U-bottom low adherence plates and cultured for 8 days. Spheroid growth was assessed by spheroid area (two-sided *t*-test). Related to Fig. 3a. **d** CAFs or 3T3 fibroblasts were cultured in 24-well plates in D2A1 conditioned medium prior to decelluarisation. D2A1 cells were plated onto the fibroblast-derived matrices and growth monitored by IncuCyte imaging (n=3 wells per condition; mean values ±SEM, 2-way ANOVA). Related to Fig. 3g. Source data are provided as a Source Data file.

Supplementary Fig. 6

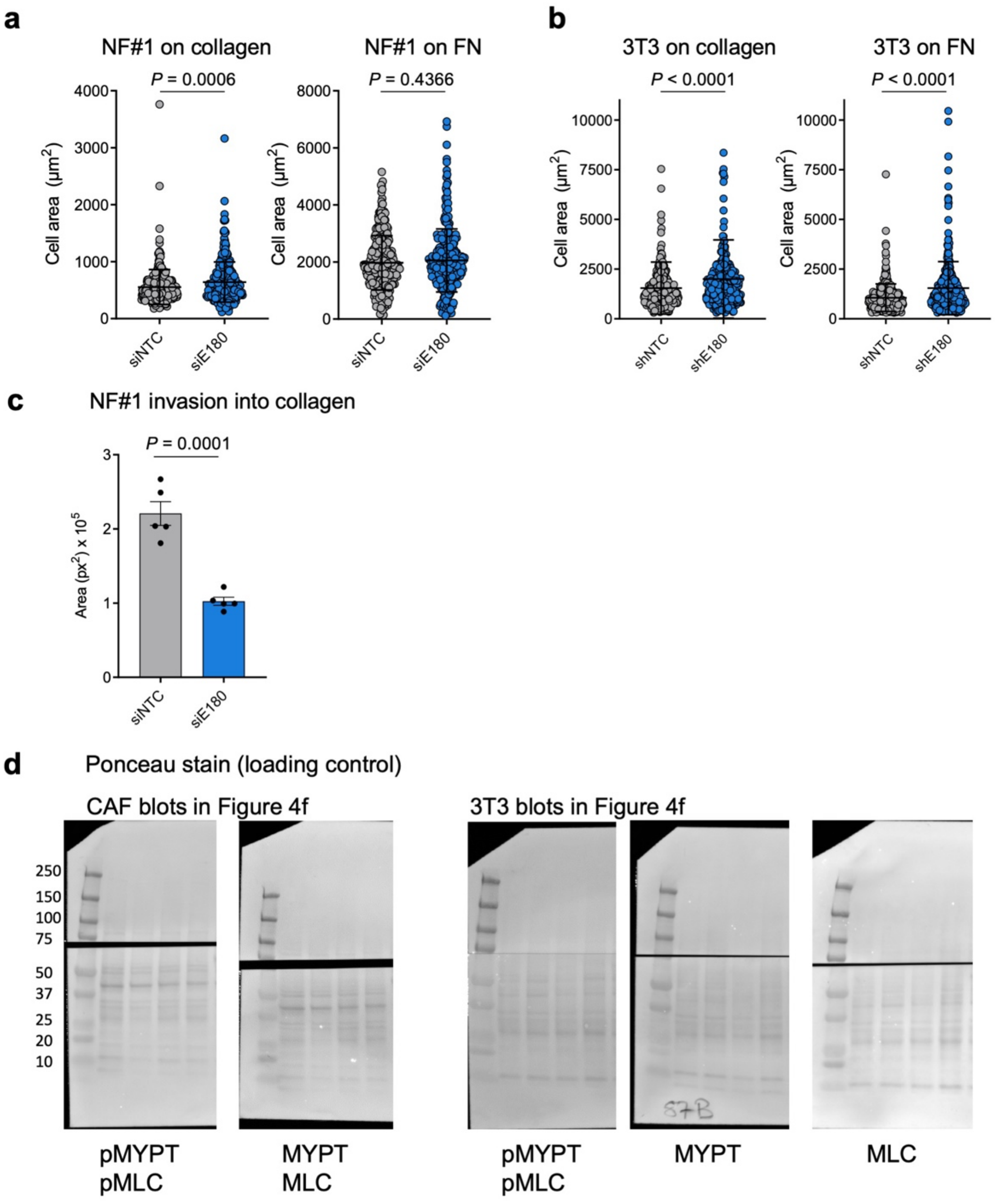

Supplementary Fig. 6 | Cell spreading on collagen or fibronectin-coated hydrogels

a,b Cell spreading (area) of siNTC and siEnd180 (siE180) NF#1 fibroblasts on collagen or fibronectin-coated stiff (50 kPa) hydrogels and shNTC and shE180-27 3T3 fibroblasts on collagen or fibronectin-coated glass coverslips, respectively, for 24 hours (n=300 cells; mean values ±SEM, two-sided Mann-Whitney *U* test). Related to Fig 4a,b. **c** Invasion of siNTC or siE180 NF#1 fibroblasts from 3D aggregates embedded in a collagen matrix after 48 hours (n=5 wells; mean values per well ±SEM, two-sided *t*-test). Related to Fig. 4d. **d** Ponceau stains (loading control) for western blots shown in Figure 4f. Source data are provided as a Source Data file.

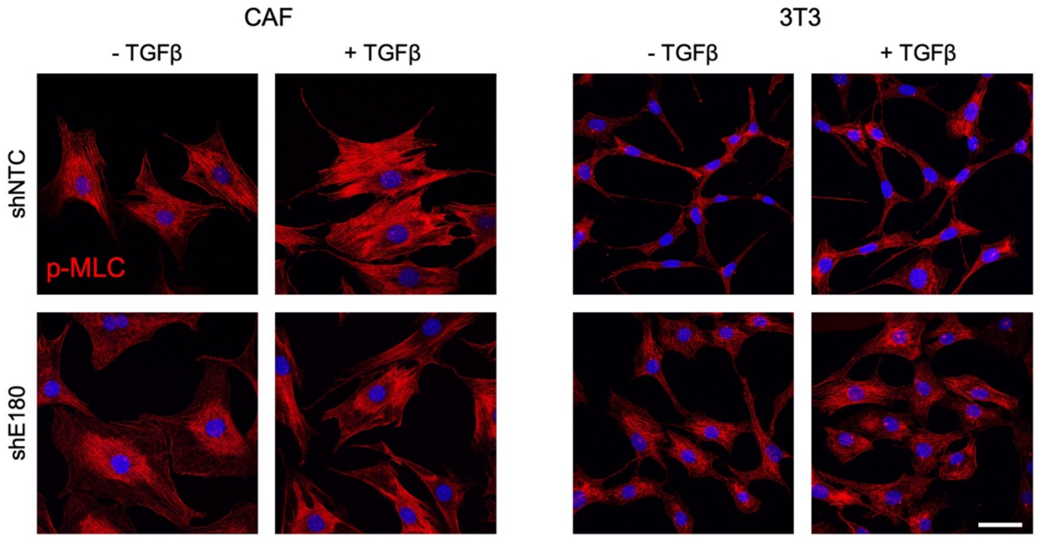

Supplementary Fig. 7 | Fibroblast contractility markers

a Cultured shNTC and shE180 transduced CAFs or 3T3s were starved overnight and then stimulated with or without TGFβ. Confocal images are replicas of those in Fig. 4g except showing only p(Ser19)-MLC (red) and DAPI stains (scale bar, 50 μm).

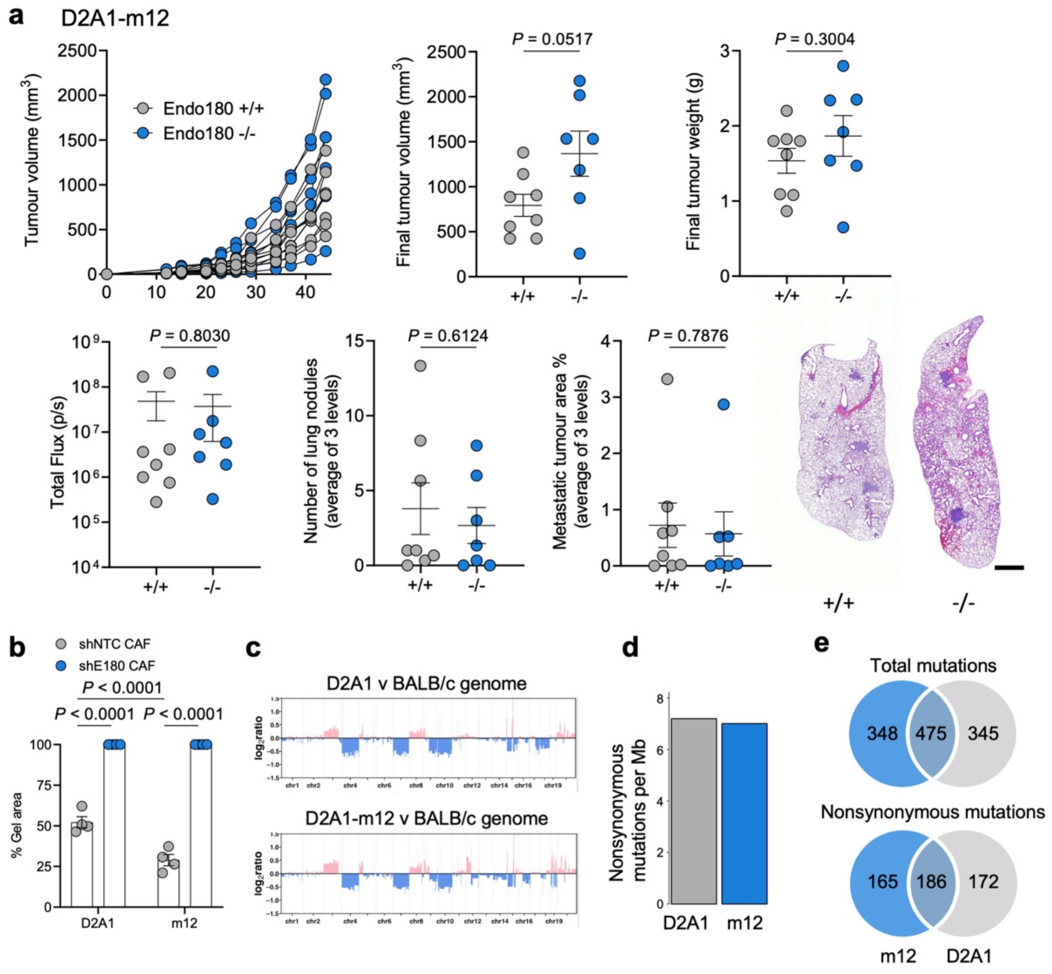

Supplementary Fig. 8 | Comparison of the parental D2A1 line and the D2A1-m12 subline
a 5 x 10^4 D2A1-m12-Luc cells were injected into the 4th mammary fat pad of BALB/c Endo180$^{+/+}$ (n=8) or Endo180$^{-/-}$ mice (n=7) and tumour volume measured twice weekly. The experiment was terminated on day 41. Shown are: primary tumour growth in individual mice, final tumour volume, primary tumour weights, quantification of metastatic burden by *ex vivo* IVIS imaging of the lungs, number of lung nodules, metastatic tumour area in the lungs and representative H&E stained lung sections (scale bar, 1 mm). All data are mean values ±SEM (two-sided *t*-test). Related to Fig. 8b. **b** Collagen contraction assay. shRNA-transduced 3T3 fibroblasts were mixed with D2A1 or D2A1-m12 tumour cells in a 1:1 ratio (each 5 x 10^5 mL^{-1}) in collagen gels. Data show % gel area after 24 hours (n=4; mean values ±SEM, two-way ANOVA). **c** Copy number variation plots (log_2 ratio) for D2A1 and D2A1-m12 cell lines using a reference BALB/c genome determined by whole-exome sequencing (WES). **d** Number of protein-coding nonsynonymous mutations per Mb of exome. **e** Venn diagrams illustrating the number of total mutations and nonsynonymous mutations in common between the D2A1 and D2A1-m12 cell lines. Source data are provided as a Source Data file.

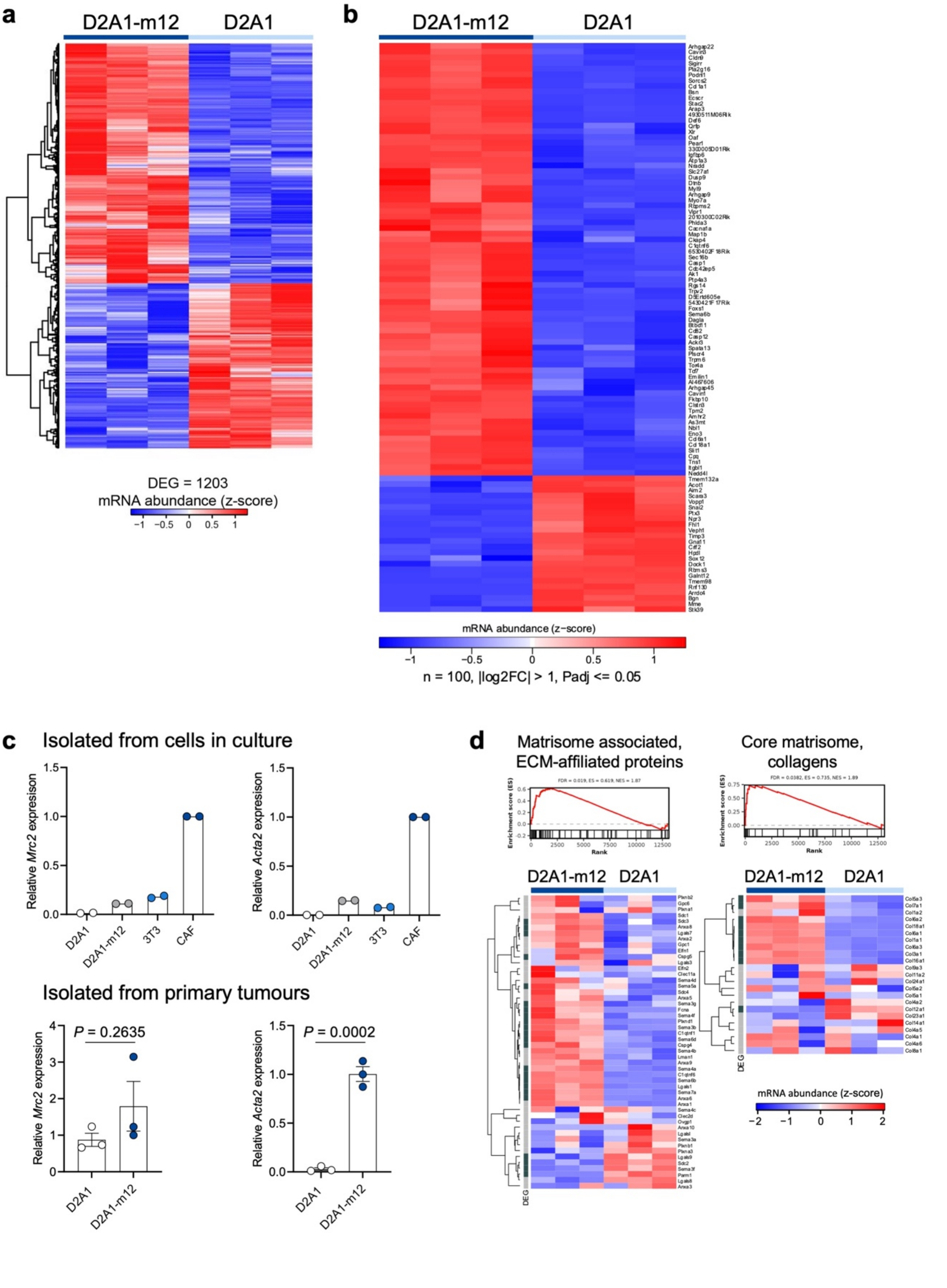
a
D2A1-m12
D2A1
DEG = 1203
mRNA abundance (z-score)
−1 −0.5 0 0.5 1
b
D2A1-m12
D2A1
mRNA abundance (z−score)
−1 −0.5 0 0.5 1
n = 100, |log2FC| > 1, Padj <= 0.05
c
Isolated from cells in culture
Relative Mrc2 expression
Relative Acta2 expression
D2A1
D2A1-m12
3T3
CAF
Isolated from primary tumours
P = 0.2635
P = 0.0002
d
Matrisome associated, ECM-affiliated proteins
Core matrisome, collagens
Enrichment score (ES)
Rank
D2A1-m12
D2A1
DEG
mRNA abundance (z-score)
−2 −1 0 1 2

Supplementary Fig. 9 | Gene expression analysis of the D2A1 line and the D2A1-m12 subline

a, b Heatmap displaying mRNA abundance of (panel a) all 1203 and (panel b) top top 100 significantly differentially expressed genes (DEGs) determined by RNA-Seq, in D2A1-m12 compared with D2A1 cells (n = 3; heatmap scale is a z-score). Threshold for differential expression was $|\log_2 FC| > 1$, and adjusted *P* value ≤ 0.05. Related to Fig. 8d,e. **c** RT-qPCR analysis of *Mrc2* (Endo180) and *Acta2* (αSMA). Upper panel, cultured tumour cells, 3T3 fibroblasts and CAFs. Lower panel, D2A1 and D2A1-m12 tumour cells isolated from 3 independent primary tumours per cell line (n=3, mean values ±SEM, two-sided *t*-test). **d** fGSEA of 'core matrisome, collagens' and 'matrisome-associated, ECM-affiliated proteins', and associated heatmaps showing the genes of the pathway (n = 3; heatmap scale is a z-score). Dark grey, significant DEGs with $|\log_2 FC| > 1$ and adjusted *P* value ≤ 0.05. Light grey, nonsignificant DEGs. Related to Fig. 8d,e. Source data are provided as a Source Data file.

Supplementary material references

1. Di Tommaso, P. *et al.* Nextflow enables reproducible computational workflows. *Nat Biotechnol* **35**, 316-319 (2017).
2. Li, H. & Durbin, R. Fast and accurate long-read alignment with Burrows-Wheeler transform. *Bioinformatics* **26**, 589-595 (2010).
3. Li, H. *et al.* The Sequence Alignment/Map format and SAMtools. *Bioinformatics* **25**, 2078-2079 (2009).
4. McKenna, A. *et al.* The Genome Analysis Toolkit: a MapReduce framework for analyzing next-generation DNA sequencing data. *Genome Res* **20**, 1297-1303 (2010).
5. Talevich, E., Shain, A.H., Botton, T. & Bastian, B.C. CNVkit: Genome-Wide Copy Number Detection and Visualization from Targeted DNA Sequencing. *PLoS Comput Biol* **12**, e1004873 (2016).
6. Wilm, A. *et al.* LoFreq: a sequence-quality aware, ultra-sensitive variant caller for uncovering cell-population heterogeneity from high-throughput sequencing datasets. *Nucleic Acids Res* **40**, 11189-11201 (2012).
7. Wang, K., Li, M. & Hakonarson, H. ANNOVAR: functional annotation of genetic variants from high-throughput sequencing data. *Nucleic Acids Res* **38**, e164 (2010).
8. Wienke, D. *et al.* The collagen receptor Endo180 (CD280) Is expressed on basal-like breast tumor cells and promotes tumor growth in vivo. *Cancer Research* **67**, 10230-10240 (2007).
9. Ponten, F., Jirstrom, K. & Uhlen, M. The Human Protein Atlas--a tool for pathology. *J Pathol* **216**, 387-393 (2008).
10. Calon, A. *et al.* Dependency of colorectal cancer on a TGF-beta-driven program in stromal cells for metastasis initiation. *Cancer cell* **22**, 571-584 (2012).
11. Davidson, S. *et al.* Single-Cell RNA Sequencing Reveals a Dynamic Stromal Niche That Supports Tumor Growth. *Cell Rep* **31**, 107628 (2020).
12. Lambrechts, D. *et al.* Phenotype molding of stromal cells in the lung tumor microenvironment. *Nat Med* **24**, 1277-1289 (2018).
