# Supplementary material for: Impairment of a distinct cancer-associated fibroblast population limits tumour growth and metastasis
Source: Nat Commun. 2021 Jun 10;12:3516. doi: 10.1038/s41467-021-23583-1 (PMC8192501; doi:10.1038/s41467-021-23583-1)
Supplement: Supplementary file 3 — Reporting Summary [file 41467_2021_23583_MOESM3_ESM.pdf]

nature research

Corresponding author(s): Clare M Isacke

Last updated by author(s): Apr 8, 2021

Reporting Summary

Nature Research wishes to improve the reproducibility of the work that we publish. This form provides structure for consistency and transparency in reporting. For further information on Nature Research policies, see our Editorial Policies and the Editorial Policy Checklist.

Statistics

For all statistical analyses, confirm that the following items are present in the figure legend, table legend, main text, or Methods section.

n/a	Confirmed	
☐	☒	The exact sample size (*n*) for each experimental group/condition, given as a discrete number and unit of measurement
☒	☐	A statement on whether measurements were taken from distinct samples or whether the same sample was measured repeatedly
☐	☒	The statistical test(s) used AND whether they are one- or two-sided<br>*Only common tests should be described solely by name; describe more complex techniques in the Methods section.*
☒	☐	A description of all covariates tested
☐	☒	A description of any assumptions or corrections, such as tests of normality and adjustment for multiple comparisons
☐	☒	A full description of the statistical parameters including central tendency (e.g. means) or other basic estimates (e.g. regression coefficient) AND variation (e.g. standard deviation) or associated estimates of uncertainty (e.g. confidence intervals)
☐	☒	For null hypothesis testing, the test statistic (e.g. *F*, *t*, *r*) with confidence intervals, effect sizes, degrees of freedom and *P* value noted<br>*Give P values as exact values whenever suitable.*
☒	☐	For Bayesian analysis, information on the choice of priors and Markov chain Monte Carlo settings
☒	☐	For hierarchical and complex designs, identification of the appropriate level for tests and full reporting of outcomes
☐	☒	Estimates of effect sizes (e.g. Cohen's *d*, Pearson's *r*), indicating how they were calculated

Our web collection on statistics for biologists contains articles on many of the points above.

Software and code

Policy information about availability of computer code

Data collection	No software was used for data collection.
Data analysis	GraphPad Prism (9.0.2 and previous), FIJI (ImageJ 1.48-1.53c), Celigo Image Cytometer (3.0.3.2-4.1.3.0), Living image software (4.3.1.0.15880-4.5.18147), Incucyte S3 (2017A Rev1 (20171.2.6415.24786)), GelCount (Version1.2.4.2), FlowJo (Version 10), QuantStudio Real-time PCR (1.0.4)

For manuscripts utilizing custom algorithms or software that are central to the research but not yet described in published literature, software must be made available to editors and reviewers. We strongly encourage code deposition in a community repository (e.g. GitHub). See the Nature Research guidelines for submitting code & software for further information.

Data

Policy information about availability of data

All manuscripts must include a data availability statement. This statement should provide the following information, where applicable:

- Accession codes, unique identifiers, or web links for publicly available datasets
- A list of figures that have associated raw data
- A description of any restrictions on data availability

The RNA-Seq data for this study (Fig. 8) have been deposited in the European Nucleotide Archive (ENA) at EMBL-EBI under accession number PRJEB36901 (https://www.ebi.ac.uk/ena/browser/view/PRJEB36901) and. The whole exome sequencing data (Supplementary Fig. 8c-e) is available under the accession number PRJEB43908 (https://www.ebi.ac.uk/ena/browser/view/PRJEB43908).
Details of publicly available datasets analysed and references to the original publications are included in the "Data availabaility" section of the manuscript.
Uncropped immunoblots are provided in the Source Data file.

Field-specific reporting

Please select the one below that is the best fit for your research. If you are not sure, read the appropriate sections before making your selection.

☒ Life sciences ☐ Behavioural & social sciences ☐ Ecological, evolutionary & environmental sciences

For a reference copy of the document with all sections, see nature.com/documents/nr-reporting-summary-flat.pdf

Life sciences study design

All studies must disclose on these points even when the disclosure is negative.

Sample size	Sample sizes for in vivo experiments were between 6 and 10 mice per group which was based on laboratory experience (Jungwirth et al. 2018 DMM) and power calculations (power 80, CI 95%, p=0.05, expected SD 15% with higher standard deviations in spontaneous metastasis assays and a SD up to 20%).<br>For in vitro studies, a minimum of triplicates was used to allow for the calculation of statistics, besides Figure 3d. Figure 1d,e, and Supplementary Figure 4a,b were only run once but included 3 technical replica. In vitro sample sizes were chosen based on previous experience for such experiments (van Weverwijk et al. 2019 Nat Coms, Jungwirth et al. 2018 DMM)
Data exclusions	A pre-established exclusion criteria for spontaneous metastasis assays are mice with intraperitoneal tumour growth as this can artificially increase the metastatic burden (2 mice in each group in Fig. 2d).
Replication	Animal experiments were repeated on at least one additional occasion with similar results except experiments shown in Fig. 1f, 1i and 1j which were conducted once. All in vitro experiments were repeated on at least one additional occasion with similar results or validated with different cell lines. Representative data from micrographs are as follows: Fig. 1a, illustrative examples of 2 cores from a 254 core tissue microarray; Fig. 2b, repeated in two independent mouse experiments, each with n=5 mice per group; Fig. 3b, repeated in >4 independent experiments with each having at least 4 replicas using siRNAs transfected and/or shRNA transduced cells; Fig. 4a/b cell shape analyses was repeated twice using fluorescent labelling, and also observed in brightfield images; Fig. 4f/g and 5b/c Western blot experiments were repeated on two additional occasions with similar results; Fig. 7a-d are representative images out of at least 6 fields of view at least one additional occasion with similar result.
Randomization	In all animal experiments mice were randomized based on individual mouse body weights at the start of the experiment. For in vitro studies, no randomization was performed as cell lines used in this study were from a single preparation with no reason to believe that the spacial location in the well impacted results.
Blinding	The investigators were not blinded for the allocation of groups during experiments. Image analysis, such as immuno-fluorescence images, spheroid area, metastasis area, and IHC staining was conducted blinded. Fully blinded animal experiments were not possible due to personnel availability to accommodate such situations.

Reporting for specific materials, systems and methods

We require information from authors about some types of materials, experimental systems and methods used in many studies. Here, indicate whether each material, system or method listed is relevant to your study. If you are not sure if a list item applies to your research, read the appropriate section before selecting a response.

Materials & experimental systems

n/a	Involved in the study	
☐	☒	Antibodies
☐	☒	Eukaryotic cell lines
☒	☐	Palaeontology and archaeology
☐	☒	Animals and other organisms
☒	☐	Human research participants
☒	☐	Clinical data
☒	☐	Dual use research of concern

Methods

n/a	Involved in the study	
☒	☐	ChIP-seq
☒	☐	Flow cytometry
☒	☐	MRI-based neuroimaging

Antibodies

Antibodies used	Information on antibodies used are provided in Supplementary Table 3.
Validation	Antibodies were obtained commercially and had been validated by the manufacture.<br>The murine Mrc2 antibody was validated in addition in siRNA and shRNA knockdown cells and knockout mice (Figure 5b). The in-house made human MRC2 mAB 39.10 was previously validated (Wienke et al. 2007 Cancer Res).

Eukaryotic cell lines

Policy information about cell lines

Cell line source(s)	4T1, 4T07, MRC5, MDA-MB-453 and NIH-3T3 cells were from Isacke laboratory stocks. D2A1 cells were from Ann Chambers laboratory stocks. E0771 cells were purchased from CH3 BioSystems. Immortalised NF#1 mouse mammary gland fibroblasts were obtained from Fernando Calvo and have been described previously. The generation of the metastatic D2A1-m2 subline has been described previously. Generation of D2A1-m12 cell line is described in the manuscript. D2A1-m12 cells are available on request.
Authentication	Human cell lines were tested using short tandem repeat (StemElite ID System; Promega).
Mycoplasma contamination	Cell lines were routinely subject to mycoplasma testing and tested negative.
Commonly misidentified lines (See ICLAC register)	No commonly misidentified cell lines were used in the study.

Animals and other organisms

Policy information about studies involving animals; ARRIVE guidelines recommended for reporting animal research

Laboratory animals	6-8 week-old female BALB/c or C57BL/6 mice were purchased from Charles River.<br>Mice with a genetic deletion in Endo180 (East et al. 2003 EMBO) on both a BALB/c and C57BL/6 background and Ub-GFP BALB/c mice were bred in-house.
Wild animals	No wild animals were used in this study.
Field-collected samples	No field collected samples were used in this study.
Ethics oversight	All animal work was carried out under UK Home Office Project Licenses 70/7413 and P6AB1448A granted under the Animals (Scientific Procedures) Act 1986 (Establishment Licence, X702B0E74 70/2902) and was approved by the "Animal Welfare and Ethical Review Body" at The Institute of Cancer Research (ICR).

Note that full information on the approval of the study protocol must also be provided in the manuscript.
